# Song Choice Is Modulated by Female Movement in *Drosophila* Males

**DOI:** 10.1371/journal.pone.0046025

**Published:** 2012-09-25

**Authors:** Alexander R. Trott, Nathan C. Donelson, Leslie C. Griffith, Aki Ejima

**Affiliations:** 1 Department of Biology, Volen Center for Complex Systems and National Center for Behavioral Genomics, Brandeis University, Waltham, Massachusetts, United States of America; 2 Career-Path Promotion Unit for Young Life Scientists, Kyoto University, Kyoto, Kyoto, Japan; Center for Genomic Regulation, Spain

## Abstract

Mate selection is critical to ensuring the survival of a species. In the fruit fly, *Drosophila melanogaster*, genetic and anatomical studies have focused on mate recognition and courtship initiation for decades. This model system has proven to be highly amenable for the study of neural systems controlling the decision making process. However, much less is known about how courtship quality is regulated in a temporally dynamic manner in males and how a female assesses male performance as she makes her decision of whether to accept copulation. Here, we report that the courting male dynamically adjusts the relative proportions of the song components, pulse song or sine song, by assessing female locomotion. Male flies deficient for olfaction failed to perform the locomotion-dependent song modulation, indicating that olfactory cues provide essential information regarding proximity to the target female. Olfactory mutant males also showed lower copulation success when paired with wild-type females, suggesting that the male’s ability to temporally control song significantly affects female mating receptivity. These results depict the consecutive inter-sex behavioral decisions, in which a male smells the close proximity of a female as an indication of her increased receptivity and accordingly coordinates his song choice, which then enhances the probability of his successful copulation.

## Introduction

The courtship behavior of the male fruit fly, *Drosophila melanogaster,* has served as a model for the study of the neural processes and computations that govern behavioral decisions. Generally, *Drosophila* males will readily initiate courtship when placed in a chamber with a conspecific female and follow a stereotyped sequence of courtship escalation [1]. First, a male recognizes a female and orients towards her. The male then taps the female on her abdomen with his forelegs, which house taste receptors. Courtship is then further escalated by male wing extensions that vibrate to produce an auditory signal. This step conveys important information to the female about male quality and is a large determinant of copulation success [2]–[3]. At this point, if the female is receptive to the male’s advances, he will lick her genitalia and immediately attempt to copulate. If unsuccessful, the male repeats the cycle, starting anew with song production [4].

The female’s role in courtship is to either accept or reject the male based upon how she perceives his courtship technique [5]. Her default state is to be unreceptive to the male. Over the course of being courted, the female decreases her locomotion, which allows the male to copulate with her [6]. The progressive decrease in female movement during successful male courtship has been thought to reflect a corresponding increase in the female’s receptivity [7]. This is further supported by studies using muted males. Females are largely unresponsive to the visual component of male courtship alone. However, when the visual courtship of muted males was paired with a playback of the acoustic male song (love song), female receptivity was restored [3] [8]. How the female integrates the visual component of courtship with the dynamics of the acoustic information, however, has yet to be fully examined.

**Figure 1:**
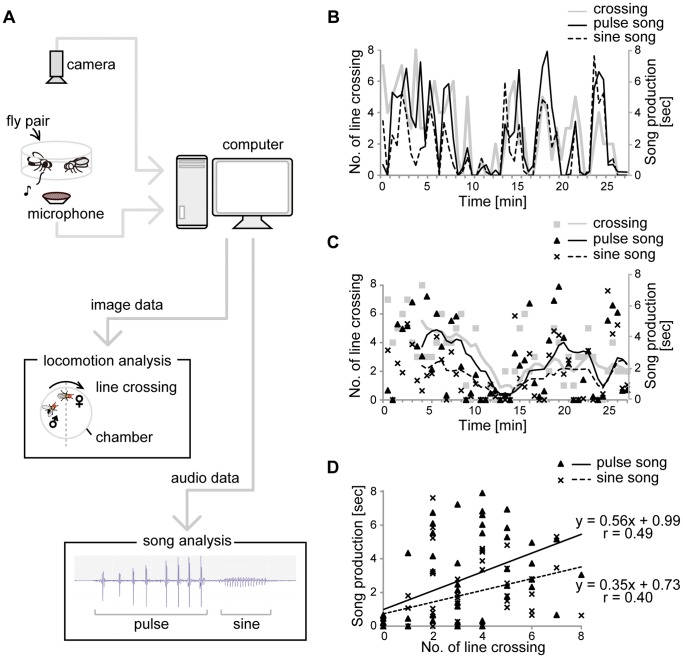
Representative recordings of female locomotion and song production in wild-type. A wild-type (WT) male and female were paired in a courtship chamber and their behaviors were recorded for 30 min. A) Schematic diagram of song analysis system. This schematic was used for all experiments examining courtship song. B) Time-course changes of the female movement, represented by numbers of line crossing, and the duration of both sine and pulse songs in each 30-second time window. Production levels of both pulse song and sine song were correlated with the female locomotion levels. C) Averaged values of the female movement and the durations of each song, calculated by 8 sliding windows of B. Averaged pulse song was preferably produced when the averaged line crossing number was over two, whereas averaged sine song production did not exceed three seconds throughout the observation. D) Song production sorted by female movement. Duration of each song is plotted against the number of line crossings, with each point representing a single 30-second window. Active female movement preferably elicited pulse song while sine song was less affected.

The love song is typically understood to represent one of the most important information transfers from the male to the female [1]. The love song is produced by male unilateral wing vibration composed of two subtypes: pulse song and sine song (Fig. 1A). The pulse song is characterized as trains of temporally punctuated “pulses” consisting of several cycles [1] [9]–[10]. The amount of time between these distinct pulses is termed the Inter-Pulse Interval (IPI) and is distinct from the carrier frequency of the signal [11]. Sine song is characterized as a ∼160-Hz humming sound [12] with its power amplitude at ∼25% of the pulse song [13]–[14]. Playback experiments showed that the pulse song increased females’ receptivity to muted males. The sine song component functions to prime the females to accept copulation, even before being paired with courting males [3] [7]–[8]. It is still unclear how the female processes the complex multimodal dynamic of the courtship ritual, especially with regards to how these different components interact (both temporally and spatially) to affect her decision to mate.

Clyde and Miesenböck [15] showed that the acoustic components of the courtship song are specified by coordinated output organized in the thoracic ganglia [15]. This suggests that local proprioception has a role in refining song characteristics via orchestration of muscle contractions [16]–[17], while the primary role of the central nervous system in courtship song is the initiation and suppression of courtship activity [15]. Two separate and methodologically distinct studies support the idea that the putative on/off neural command region involves a site integrating multiple sensory modalities [18]–[19]. Broughton *et al.* [18] specified the lateral posterior protocerebrum as the courtship decision region, though it was likely not the only courtship-relevant site of sensory integration. As discussed by Clyne and Miesenböck [15], data regarding central and motor control over production of pulse song versus sine song likely adheres to one of two models: (1) the brain signals either sine or pulse song directly, each through its own separate input or (2) the brain has an “indiscriminate” signal to sing with no song type specified, and a separate “pure” signal controls the specific parameters of the motor pattern that bias the musculature towards a distinct song type. In order to understand how the song production decision is made (that is, pulse song versus sine song) and its relationship to sensory inputs, we examined how male courtship song is produced in the context of female behavior and how it is affected by the absence of olfactory input.

## Results

### Female Locomotion Determines Pulse and Sine Song Proportions

Males choose the subtype of the love song to produce in the context of female behavior. The locomotor activity of a female and the song profile of a male were analyzed during an observation period of 30 consecutive minutes (Fig. 1A). Locomotor activity was measured by recording the number of times the female crossed a line laid down the middle of the chamber, and the male’s total time spent singing either pulse or sine song was analyzed for each 30-second time window within the observation period. Both raw (Fig. 1B) and smoothed (Fig. 1C) activity records showed that production levels of both pulse song and sine song were positively correlated with the female’s level of locomotion. As female activity increased, total song production also increased. Pulse song was produced preferentially when the female crossed the line more than twice in the 30-second bins, whereas the averaged sine song duration reached a maximum of ∼3 seconds per 30-second window (Fig. 1C). When song production levels were plotted as a function of the number of line crossings, active female movement preferentially elicited pulse song, while sine song was less sensitive to movement levels (Fig. 1D, difference in slope, ANCOVA, *p*<0.05). As the female gradually reduced her locomotion over time, the male shifted his courtship song from pulse-dominant to an equal blend of sine-and-pulse. In general, males initiate courtship with loud pulse song when the female is not in close proximity. But once the female reduces her locomotion and gets closer, indicating an increase in her receptivity to copulation, the male shifts his song proportion to the sine song.

**Figure 2:**
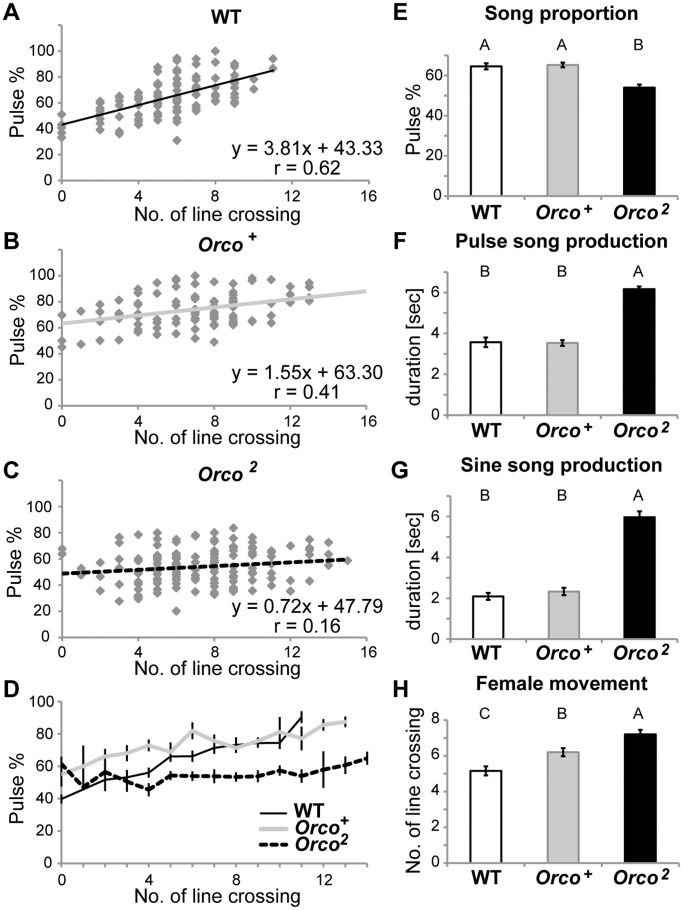
Song profile of wild-type and olfactory mutant males. In order to investigate how the song choice is controlled, proportion of pulse song among total song production (Pulse %) was analyzed in multiple courtship sessions (>5 min, N >5 each) and plotted as a function of female movement. Each point represents a single 30-second window of courtship. A) Wild-type. The proportion of pulse song (Pulse %) is tightly correlated with the number of line crossings of the target female. B) Genetic control strain *Orco^+^* and C) olfactory mutant strain *Orco^2^*. D) Mean Pulse % plotted against each female movement window. The olfactory deficient males *Orco^2^* produced consistent Pulse % regardless of the female movement and showed significantly lower proportion of pulse song compared to the genetic control *Orco^+^* and wild-type when the female was actively moving, indicating that olfactory cues provide information related to female activity and/or the male’s proximity to the female. E) Mean Pulse %, F) pulse song production, G) sine song production and H) female movement throughout. The *Orco^2^* males produced significantly higher levels of both sine song and pulse song and the courted female showed higher levels of movement. Different letters signify significant differences between groups (p<0.05).

How does the male dynamically change his song strategy? We analyzed the song proportion of multiple courtship sessions from multiple pairs of wild-type males and females and confirmed that the proportion of pulse song in total song production (Pulse %) was significantly correlated with the number of line crossings of the target female (*r = *0.6243, *p*<0.001, Fig. 2A). The equal weighting of pulse and sine levels at low levels of female movement (Pulse % ≈ 50, when line crosses ≤4) suggests a song regime in which sensory signals provide comparable or indiscriminate stimulation for pulse and sine songs. The fact that an increase in female movement is associated with an increase in the Pulse % implies that female movement may provide a pure-pulse-stimulatory signal that modulates the song characteristic to become a pulse-dominant one.

It is still possible, however, that the observed correlation occurs instead because the female dynamically adjusts her locomotion in response to the song proportion such that male’s song profile is not a direct consequence of female movement. In order to examine this possibility, we paired a male with a non-moving headless female. The averaged Pulse % of males toward such immobile females was as low as 31.06±2.99% (Fig. S1A), showing that female movement levels, not simply the presence of a female, caused the male to weight his song production towards pulse song.

### Temporal Relationship between Distance and Song Bout Onset

An object-tracking program was developed to calculate the distance between the two flies in each frame of the video captured while recording (30 frames per second) to see the effects of distance on song production. The average distance for the windows spanning 3 seconds before to 3 seconds after the onset of each bout of a given type of song was measured. Figure S2 shows that the distance between the flies is shorter in the windows of time just before the onset of sine song production (gray line) as compared to those just before the onset of pulse song production (black line). This suggests that the probability of sine song being produced is inversely proportional to the distance between the two flies. This can be thought to reflect the function of the putative song modulatory pathway in the male, by which an increase in distance to the female stimulates pulse song production while closer proximity to the female activates sine song, though not exclusively.

### Olfactory Control of Song Choice

How does a male detect the female’s movement and/or his proximity to her and dynamically change his behavior accordingly? In order to assess the contribution of the olfactory system to the song choice, we examined the song profile of olfactory deficient *Orco^2^* males that have a null mutation in the *Orco* gene [20]. The *Orco* gene, previously reported as *Or83b*, mediates localization of chemoreceptor ion channels to the membrane of odorant receptor neurons (ORNs). Because of the gene’s broad expression throughout ORNs, its deletion has the capability to deprive the flies of olfactory input [20]. Figure 2C shows that the *Orco^2^* mutant males produced uniform Pulse %, regardless of female movement, rendering the correlation between the Pulse % and the numbers of female line crosses non-significant (*r = *0.1606, *p*>0.05). The genetic background control line, *Orco^+^*, showed a song profile similar to that of wild-type (Fig. 2B, *r = *0.4055, *p*<0.001). *Orco^2^* males produced a significantly lower proportion of pulse song compared to control at movement levels greater than four crossing per 30-second window (Fig. 2D and Table S1 for each significance test after ANOVA). The averaged Pulse % throughout the observation also showed an overall reduction of pulse song proportion for *Orco^2^* males (Tukey HSD, *p*<0.001, Fig. 2E). These results suggest that the dynamic adjustment of song proportion in response to changing female movement requires access to olfactory cues.

These different strains of flies vary not only in aspects of their song proportion but also in their general song production. Olfactory mutant males produced significantly higher levels of both sine song and pulse song (Tukey HSD, *p*<0.001 for both song types, Fig. 2FG, Fig. S3 and Table S2). Therefore it is unlikely that the reduced levels of Pulse % in the *Orco^2^* males resulted from an inability to produce pulse song.

Females were observed to move at significantly higher levels when courted by the *Orco^2^* males (Tukey HSD, *p*<0.05, Fig. 2H). Based on the positive correlation between the Pulse % of wild-type males and the level of female locomotion (Fig. 2A and Fig. S1), if male song proportion drives changes in female movement, females would be expected to move less when stimulated with the consistent and relatively low Pulse % of the *Orco^2^* males. This was not the case, however, and we conclude that the direction of temporal causality is female to male; the female movement levels determine male’s song choice and not the other way around.

**Figure 3:**
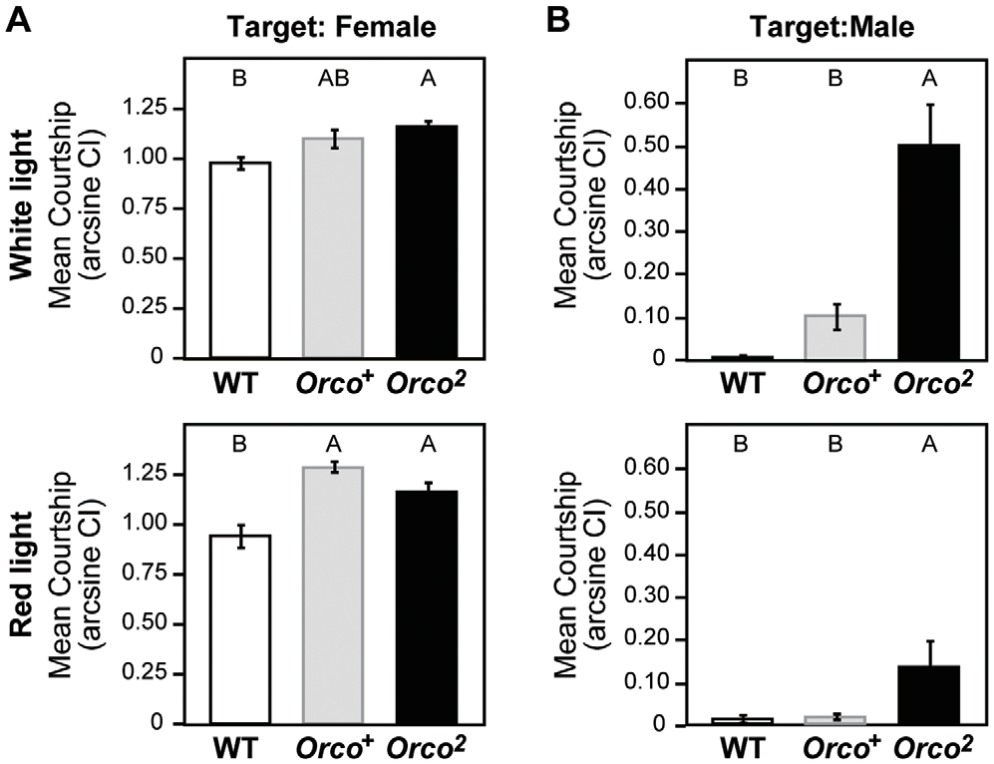
Courtship assay. Courtship vigor of wild-type and olfactory mutant males was measured toward immobile wild-type females (A) or males (B) under white (top panels) or dim-red light (bottom panels) conditions. 15∼28 males were tested for each genotype and experimental condition. Different letters signify significant differences between groups (p<0.05).

We also performed courtship assays using immobile target flies to measure courtship motivation of the males independent of female responses in a given time. As shown in Figure 3A, the courtship level of *Orco^2^* males toward immobile females was significantly higher than wild-type (WT, Tukey HSD, *p<*0.05) but not different from the *Orco^+^* genetic control) under white light (Tukey HSD, *p*>0.05. In dim-red light, where a fly was unable to utilize visual cues, the *Orco* ^2^ mutant males showed a level of courtship indistinguishable from the *Orco^+^* control, confirming that the *Orco^2^* mutant males were capable of performing normal levels of female courtship (Tukey HSD, *p*>0.05). On the other hand, *Orco^2^* males showed high courtship toward other males compared to both the genetic control *Orco^+^* and wild-type in both white light and red light conditions, suggesting an impaired ability to discriminate the sex of the target fly (Fig. 3B). These findings are consistent with a previous study on an olfactory-deficient mutant *olfD*, in which the *olfD* males showed normal courtship vigor to a virgin female but lost the ability to detect male-derived pheromone on a mated female [21]. The level of male-directed courtship by *Orco^2^* males was still less than female-directed courtship, probably because of the intact function of other chemosensory inputs (e.g. gustation can provide information about the sex of the target [22]). All results support the idea that *Orco^2^* males have a deficit only in olfactory ability, indicating that the impaired control of song proportion seen in the mutant males resulted from lack of olfactory input.

Supporting the idea that proper olfactory cues are crucial for plastic control of song choice, an impaired Pulse % shift was also found in wild-type males when they were paired with immature females. Immature females, who have just eclosed from their pupal cases, are known to contain only precursors of pheromone hydrocarbons and therefore not to emit a mature female smell [23]–[24]. As shown in Figure S1B, the males produced courtship song with consistent Pulse % (average 55.27±1.86%) regardless of female movement levels, indicating that pheromonal information plays an important role in coordinating song proportion with locomotion levels of the target female. Intriguingly, young females were found to be very active, providing no data points lower than three line crossings per 30 seconds (Fig. S1B) and the average number of crossings was 8.19±0.38, which was significantly higher than that of mature females (5.47±0.26, *p*<0.001, Fig. 2A). As a result, the Pulse % shift of the males paired with the immature females was biased to have a negative slope (Fig. S1B), in contrast to our previous observations. It is worth noting that wild-type males courting immature females show a remarkably similar song profile to *Orco^2^* males courting mature females (compare Fig. S1B to Fig. 2C). This strongly supports the conclusion that olfactory cues mediate the dynamic adjustment of song proportion observed when control males court mature intact females.

### Reduced Copulation Success of Olfactory Deficient Males

**Figure 4:**
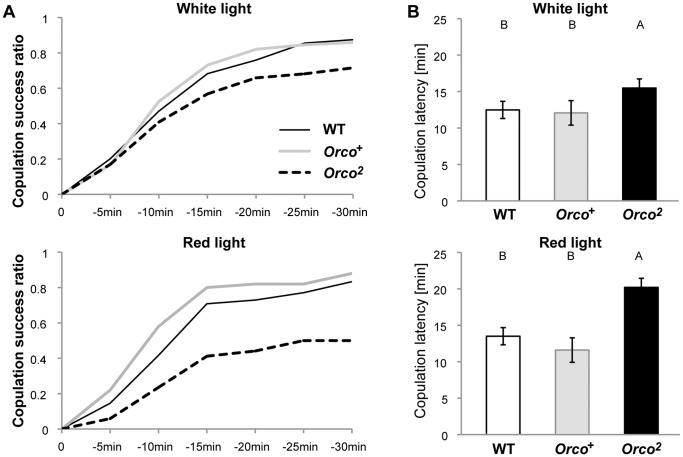
Copulation success of wild-type and olfactory mutant males. In order to investigate how the song choice affects female receptivity, intact wild-type females were paired with WT, *Orco^+^* or *Orco^2^* males and numbers of successful copulation were counted under white (top panels) or dim-red light (bottom panels) conditions. A) Cumulative proportion of pairs reaching successful copulation over time. B) Copulation latencies of males of each genotype. ≥38 males were tested for each genotype and experimental condition. Different letters signify significant differences between groups (p<0.05).

The number of line crossings was significantly higher in the females paired with the olfactory mutant males (Tukey HSD, *p*<0.05, Fig. 2H). Considering the absolute amount of pulse song of the *Orco^2^* males was unimpaired (Fig. 2F), this implies that there are additional criteria that females use to assess the quality of male courtship. In order to investigate the reproductive success of each group, the number of successful copulations during 30 min was counted for wild-type, *Orco^+^*, and *Orco^2^* males. As shown in Figure 4, the olfactory deficient *Orco^2^* males had a significantly lower copulation success rate compared to the controls, indicating that intact olfactory function played a critical role in successful copulation. Assuming the only perceptible difference between these groups of males as far as the female is concerned is their ability to modulate Pulse % in response to her movement (no difference was found in the acoustic properties of the strains’ song [Trott, data not shown]), this argues that females respond to the male’s ability to properly coordinate song choice.

It is possible that the reduced success of the *Orco^2^* males reflects additional deficits that were not detected in this study. In order to test the importance of modulating song profile in courtship success, we repeated the copulation assay using wingless males. As shown in figure S4, when their wings were clipped off to block song production, control males lost their copulation advantage. During the extended observation period of 60 min, no more than 20% of males (each N = 24) of any genotype succeeded in copulation and there were no significant differences in the copulation latencies. The copulation advantage of intact control males therefore likely derives from behaviors pertaining to song production. Because song production was neither reduced (Fig. 2FG) nor acoustically aberrant among *Orco^2^* males, their reduced success is suggested to be a result of their failure to modulate Pulse % in response to female movement.

## Discussion

The influence of sensory processing on male courtship behavior is multimodal in nature, involving visual, gustatory, olfactory, and mechanosensory inputs [25] [25]. While extensive studies have been done on the genetic and anatomical control of initiation and maintenance of courtship [26]–[28], less attention has been given to how the courtship quality is controlled to maximize successful copulation in *Drosophila*. It is broadly well-known that song structure plays a critical role as an indicator of male quality for female mate choice in songbirds [29], frogs [30]–[31], crickets [32] and many other species. In this study, we demonstrated that the *Drosophila* male dynamically adjusts the proportion of its song subtypes, pulse song or sine song, in response to changing female movement (Fig. 2A). When a female moved at low speed, indicated by less line crossings, the song ratios of pulse and sine were maintained at nearly equal levels. As female movement increased, the song dynamics became pulse-dominant. The distance trace assay for song onset revealed that sine song tended to be produced when the male got closer to the female while the opposite was true for pulse song (Fig. S2). Considering that the level of female movement serves as an indicator of the female’s mating receptivity, these results imply that the male produces pulse-dominant song when the female is still unreceptive and then shifts to an equal blend of sine-and-pulse as the female gradually increases her receptivity and accordingly reduces her locomotion.

We also report that olfactory information is required for this song modulation. Olfactory mutant males, who had impaired pheromone discrimination, were unable to modulate song proportion in response to female movement (Fig. 2, 3). Since there is no reduction of total song amount observed in these males (Fig. 2FG), olfactory input was not critical for song production itself; instead, it played an important role in flavoring the song properties. These mutant males also showed low copulation success (Fig. 4), indicating that the male’s ability to dynamically modulate song choice influences the female’s mating decision. It is still unclear, however, how olfactory information modulates song choice. The olfactory mutant males’ song profile was indistinguishable from the controls’ when the female activity was low; however, the mutant males failed to increase the proportion of pulse song in response to increases in female locomotion (Table S1). This observation implies that olfaction plays a critical role in coordinated pulse song production, shifting the song property from pulse = sine as the default proportion to pulse>sine when the female moves away. In this case, considering the low volatilities of *Drosophila* pheromones in general [23], an “OFF” response of the sensory neurons caused by the “reduction” of odor intensity might signal an increase in the distance to the female target and stimulate the male to produce the pulse-oriented song. It should be noted, however, that the opposite scenario is still possible, in which the default song proportion has been biased to pulse-dominant (pulse>sine) and then strong odor inputs from the female, when she is in close proximity, specifically stimulate sine song production and accordingly suppress the ratio of pulse song to the level of sine song (pulse = sine). When a male was paired with a motionless female and noticed her presence, the male performed vigorous courtship to the female in close proximity [22] and produced sine-dominant song (Fig. S1A). This result predicts that strong odor inputs from the target female specifically stimulated sine song production, favoring the latter model. Further studies would be needed to discriminate between these models of song modulation.

This study also provides new insights on how male courtship is controlled redundantly by multiple modalities [33]–[34]. Normal, or even higher, levels of female courtship in the olfactory mutant *Orco^2^* (Fig. 3A) indicate a function for cross-modal compensation, in which the balance of sensory saliency is modulated according to availability (i.e. chronic absence of olfactory input in *Orco^2^* males may be compensated by increases in the weight of signals from preserved modalities, probably vision or mechanosensation in this case [33] [35]). The increase in total song in *Orco^2^* appears to be stimulated when the target female is actively moving (Fig. S3). The neural circuit from the non-olfactory modality, which may play an expanded role in stimulation of courtship in the *Orco^2^* males, is activated in a less controlled way by active female movement and conveys an indiscriminant signal that triggers general song production but doesn’t designate song type, causing *Orco^2^* males to sing in a non-strategic way (Fig. 2). Further investigation on the neural processing in the sensory integration regions [18]–[19] of this mutant males will open up a novel avenue of research on molecular mechanism for cross-modal plasticity [36], in which sensory deprivation in one modality develops the remaining modalities in an experience-dependent manner. The previous studies using the *Orco* mutants have explained all behavioral differences, including locomotion defects [38] and enhanced longevities [39], as consequences of their olfactory impairments. Therefore, we interpret the findings from the *Orco^2^* males as an indication of how olfaction contributes to the observed courtship phenomena. However, given the possibility of cross-modal compensation and/or a potential non-specific effect of the *Orco* mutation throughout development, we acknowledge the possibility that the observed phenotypes of these mutants might have resulted from non-olfactory control of the behaviors.

## Materials and Methods

### Fly Strains

All fly lines were raised on cornmeal-sucrose-agar food containing inactivated yeast. The flies were kept in a 25°C incubator with a 12 hr Light/Dark cycle. Adult male virgin flies were collected within 4 hours of eclosion using CO^2^ anesthesia, and aged for 4–6 days in individual test tubes. Only males with fully intact wings were used in the experiment. Virgin male and female *Canton-S* (*CS*) flies were collected for use as courtship objects, and kept communally in sex-specific vials. *Orco^+^* was used as a genetic control for the *Orco^2^* olfactory deficient mutant, and *CS* served as a wild-type control. The two *Orco* lines are generated through ectopic insertion of a gene-targeting construct [20]. For the *Orco^2^* olfactory deficient mutant this insertion interrupts the *Orco^2^* gene, and for the *Orco^+^* controls it is inserted into a random location on the 3^rd^ chromosome; the lines otherwise share the same genetic background.

### Song Recording

Courtship song was recorded from single male and female pairings. All audio recording was accompanied by video recording to allow comparison between song production and courtship events. All recording conditions used an Aktogen Courtship Song Chamber made of a 3 mm tall cylindrical chamber with a diameter of 12 mm on bottom and 14 mm on top and equipped with a movable isolation barrier to control when the flies could interact. The chamber rests on top of a microphone platform housing two specialized Aktogen microphones that feed into an amplifier. A video camera was situated just above the transparent chamber. Video and audio were recorded simultaneously into a computer using QuickTime Pro (Apple, USA). All recordings took place in a semi-anechoic soundproof room. The test male and his courtship target were loaded separately into divided regions of the chamber. After removing the barrier between the flies, recording began immediately after the commencement of song production. The male was allowed to court the female target for a fixed amount of time (15 minutes, unless otherwise stated). In the event that copulation occurred, recording was terminated. Within a given experimental paradigm, the goal was to record approximately 1 hour of total courtship per strain. Because of differing baseline intensities and rates of copulation success, this required a different number of flies per strain, though generally as little as 5 to 10 pairs of flies were sufficient. We controlled for potential environmental or circadian effects in the following ways: All recording days began ∼4 hours after the start of the light cycle; For experiments involving comparisons across multiple strains, all strains were included on a given day and tested in an interleaved order; Before each pair was tested, the recording chamber was dismantled, cleaned with 70% ethanol, dried, and reconstructed; When needed, a portable heating device was used in between trials to keep the temperature around the chamber near 25°C. Circadian phase at the time of recording was kept consistent across groups.

### Song Analysis

After recording, audio extracts of the song files were pre-processed using Amadeus Pro (version 1.5.4, HairierSoft). This involved first employing a high-pass filter to remove all frequencies between 0 and 120 Hz to eliminate the noise from the computer and most of the periodic noise from outside of the room. Following the high-pass filter, the Suppress Noise tool on Amadeus Pro was used. This tool uses the frequency profile of a user-selected portion of pure background noise to construct a filter that is then applied to the full audio file. While relatively little processing was needed to improve the signal associated with pulse song, sine song was often difficult to hear behind the noise. These manipulations greatly enhanced the signal quality of sine song, making it clearly audible and distinguishable. Pre-processed songs were scored in two ways: by hand using Amadeus Pro and in an automated fashion using a custom MATLAB 2008b (Mathworks, Inc) script. In general, pulse and sine are clearly distinguishable when there is little or no background noise (as was the case for pre-processed songs); in such cases, either listening to the sound file or looking at the sound trace was sufficient to identify song. When the identification was less absolute, the decision of whether to score the sound in question was based on spectral composition. The criteria included the presence of a prominent and at least somewhat narrow (no more than 20–30 Hz wide) spike in the intensity of the sound at a frequency between 125 and 200 Hz.

The automated analysis employed similar quantitative criterion for identifying sine song; pulse song was identified by searching for repetitive sequences of quick bursts in the waveform amplitude. More specifically, to identify sine song the algorithm first separated the song file into discrete 50 ms windows. For each window, FFT was used to obtain the power spectrum of the sample; the window was then scored as sine song if the sample contained enough acoustic energy and a sufficiently high fraction of the total acoustic energy occurred between 120–200 Hz. The algorithm scored pulse song by first identifying putative pulses and then grouping trains of pulses into bouts. An individual time point was defined as being part of a pulse if the absolute z-score of the waveform at that time was above a given threshold. The z-score was calculated using the mean and standard deviation of the data window starting 20 ms prior and ending 5 ms prior to the time point in question. Pulses were only labeled if the surrounding sample contained enough acoustic energy. The exact values of the above criteria (which were constant throughout the analysis) were chosen to minimize any obvious tendencies for the algorithm to miss-label the song files. We verified that our results were not a product of qualitative or subjective judgments during hand scoring by confirming that the results of analyses performed on the song data from the two separate scoring methods did not differ meaningfully. The analyses in this report used the data collected from hand-scoring, since this method ultimately included a more thorough set of scoring criteria.

When analyzing song profile, we excluded the 30-second windows of courtship where less than 2.5 seconds of song were measured to avoid potential artifacts. Pulse % was calculated as the fraction of time spent singing pulse song during a given window over the total time spent singing. When analyzing production of specific song components, time spent singing each song was reported for each full 30-second window regardless of the total amount of song produced.

### Line Crossing Assay

Female movement was quantified using the video captured during the courtship recording. For each full 30-second window, movement was quantified as the number of times the female crossed the vertical line that was drawn to divide the chamber in half. When the female was about to cross the line but immediately turned around, crossings were counted only if the female head reached a designated area.

### Object Tracking

For a more complete analysis of the relationship between song profile and distance, an object-tracking program, FlyPairTracker was created using MATLAB. The FlyPairTracker compared each frame of video to the average of the full video file and labeled discrete pixel clusters whose absolute differences were sufficiently greater than the background. The program also recognized flies based on their sizes and the inherent assumption that objects move in continuous paths. At each frame, distance was measured as the Euclidean distance between the flies’ centers of mass. Distance was calculated by normalization of the distance in pixels separating two distinct structures in the chamber, the length of which changed as a result of slight differences in camera positioning during recording. MATLAB was also used to automate the song annotation process as described above in order to examine the relationship between song production and distance with higher temporal resolution. The program was designed to objectively identify segments of the song file as pulse, sine, or (by default) silence and was used to find the exact time points at which the male produced either pulse or sine song. For every separate bout of a given type of song, the distances separating the flies from 3 seconds before to 3 seconds after the onset of each bout were collected. We then averaged these distance traces for each type of song to provide an intuition for how male/female proximity is related to song selection.

### Courtship Assay

On the day of experimentation, the courtship object flies were decapitated with micro dissection scissors just before use. Decapitated flies that were not standing after anesthesia recovery were excluded. All experiments were conducted beginning at ZT1 in a Harris environmental room (25°C, 70% humidity). Male test flies were transported into individual courtship observation chambers (8 mm diameter, 3 mm depth) using a mouth aspirator. The flies were allowed to acclimate for 5 min. A single decapitated fly was then introduced to each male and the interaction was immediately video recorded for 10 min (Logitech webcam using QuickTime Player). Four different experimental conditions were tested: male courting female or male courting male in white light or dim red light. No single flies were used in subsequent experiments. Video was hand scored to determine the total amount of courtship that occurred during the 10 min trial. Courtship was defined as described previously [37]. The courtship index (CI) was calculated as the total amount of time the males spent courting per 10 min period. Flies that successfully mated were excluded from the experiment.

### Copulation Assay

Using the same observation chambers as courtship assay (8 mm diameter, 3 mm depth), a male was paired with an intact CS female in a courtship observation chamber under white lights or dim-red lights (which limit visual information). When necessary, wings of the test males were clipped off with micro dissection scissors on the day before the test. The copulation latency, the time lag after pairing until successful copulation, was recorded. The copulation success ratio was the number of copulation successes at each given time point as a fraction of total observed number. A maximum latency value, 30 min or 60 min, was given when no copulation was performed during 30 min or 60 min observation, respectively.

## Supporting Information

Figure S1 **Proportion of pulse song of wild-type males toward decapitated motionless female (A) or intact immature female (B).**
(TIF)

Figure S2 **Average of the distance separating the target female from the wild-type male relative to each song onset.** Pulse song is plotted in black; sine song is plotted in gray.Thick lines show mean distance; dashed lines show SEM.
(TIF)

Figure S3 **Average total song production plotted against each female movement window.**
(TIF)

Figure S4 **Copulation success of wild-type and olfactory mutant males when their wings were amputated.** Same convention as Figure 4 in the main text. Different letters signify significant differences between groups (p<0.05).
(TIF)

Table S1 **Song profile of WT,** ***Orco^+^*** **and** ***Orco^2^*** **.**
(PDF)

Table S2 **Test results for total song production (Fig. S3).**
(PDF)
