# Supplementary figures and images for: Song Choice Is Modulated by Female Movement in Drosophila Males
Source: PLoS One. 2012 Sep 25;7(9):e46025. doi: 10.1371/journal.pone.0046025 (PMC3458092; doi:10.1371/journal.pone.0046025)

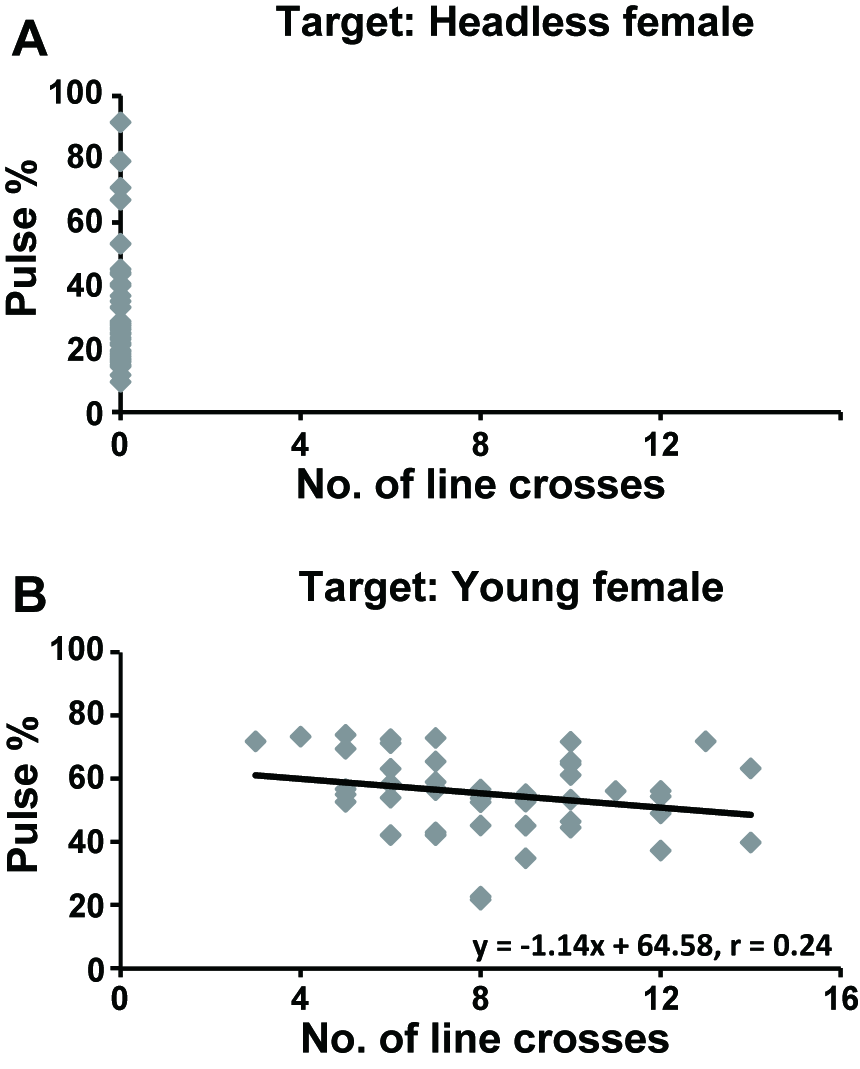

Supplement: Figure S1 — Proportion of pulse song of wild-type males toward decapitated motionless female (A) or intact immature female (B). (TIF) [file pone.0046025.s001.tif]

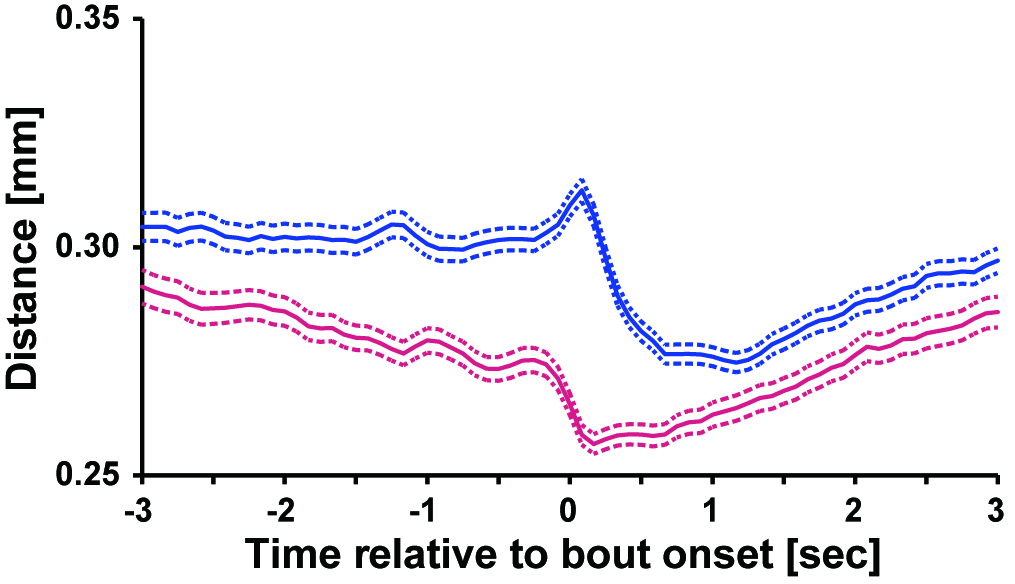

Supplement: Figure S2 — Average of the distance separating the target female from the wild-type male relative to each song onset. Pulse song is plotted in black; sine song is plotted in gray.Thick lines show mean distance; dashed lines show SEM. (TIF) [file pone.0046025.s002.tif]

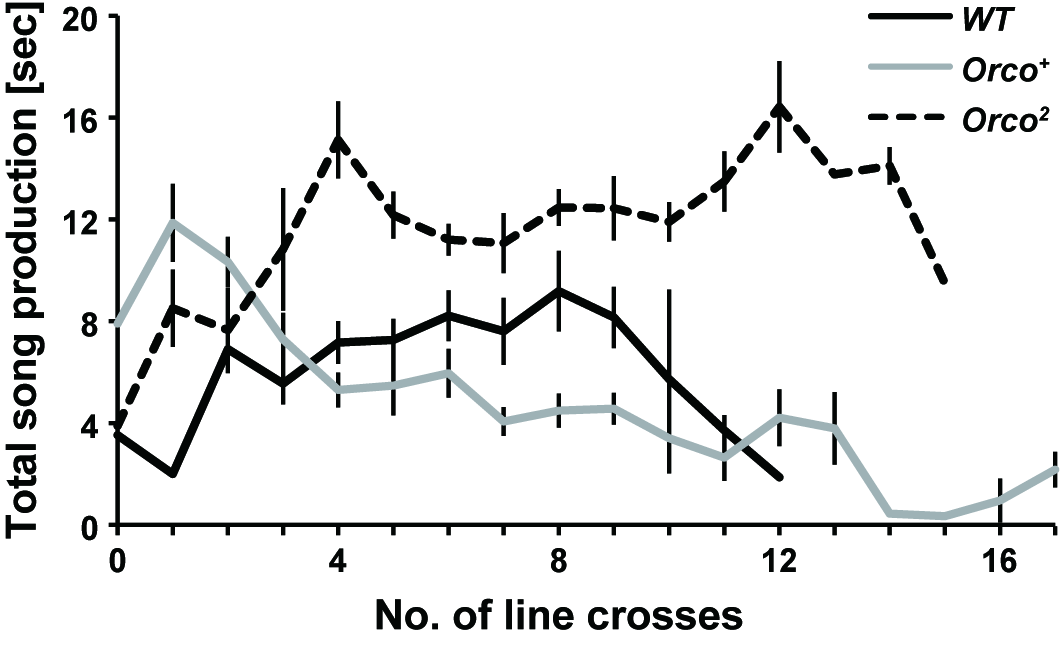

Supplement: Figure S3 — Average total song production plotted against each female movement window. (TIF) [file pone.0046025.s003.tif]

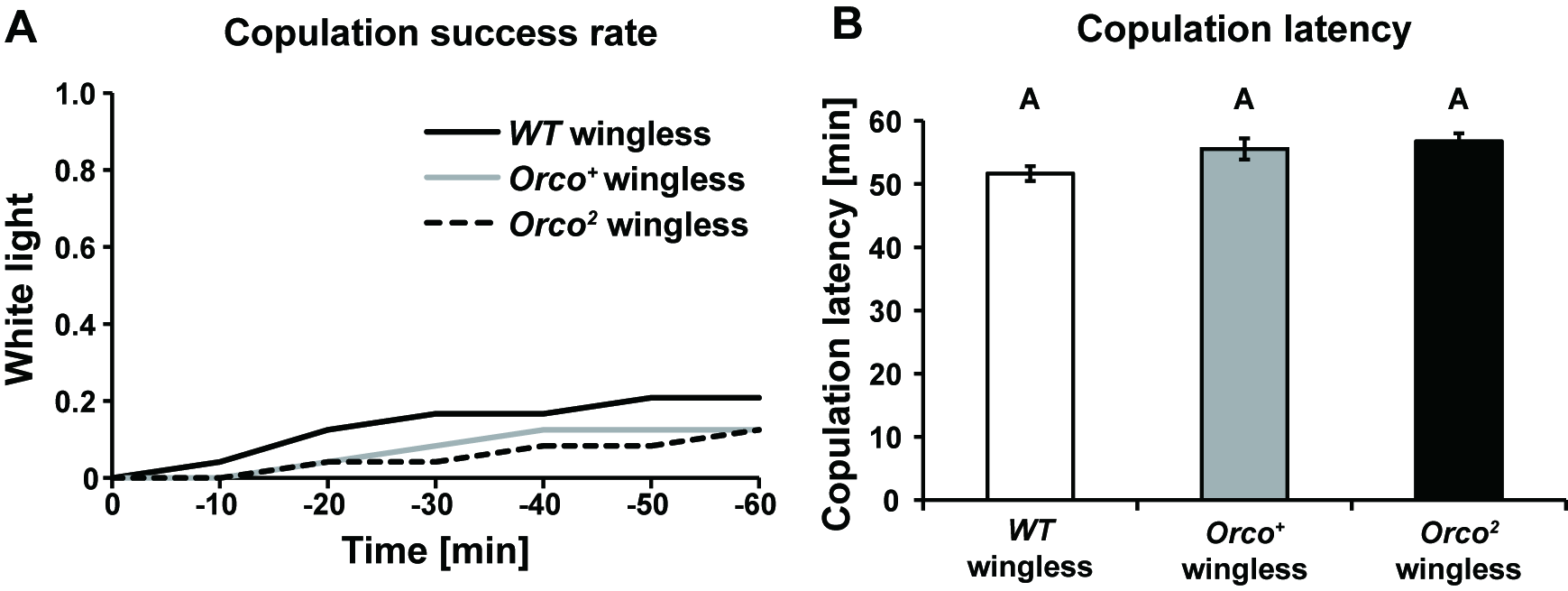

Supplement: Figure S4 — Copulation success of wild-type and olfactory mutant males when their wings were amputated. Same convention as Figure 4 in the main text. Different letters signify significant differences between groups (p<0.05). (TIF) [file pone.0046025.s004.tif]
