# Supplementary material for: Song Choice Is Modulated by Female Movement in Drosophila Males
Source: PLoS One. 2012 Sep 25;7(9):e46025. doi: 10.1371/journal.pone.0046025 (PMC3458092; doi:10.1371/journal.pone.0046025)
Supplement: Table S1 — Song profile of WT, Orco+ and Orco2 . (PDF) [file pone.0046025.s005.pdf]

TableS1: Song profile of WT, *Orco⁺ and Orco²*

line crossing	mean % Pulse WT			*Orco*$^+$			*Orco*^{2}			ANOVA	ttest result WT	*Orco*$^+$	*Orco*^{2}
0	39.77	±	2.80	55.01	±	7.53	61.45	±	4.46	$p < 0.05$	B	A	A
1				60.03	±	12.74	47.16	±	0.57	NS			
2	51.68	±	2.70	66.10	±	4.65	56.50	±	11.86	NS			
3	53.17	±	4.62	68.06	±	4.98	50.56	±	6.30	NS			
4	56.05	±	3.10	72.89	±	3.60	45.50	±	4.04	$p < 0.05$	B	A	C
5	66.09	±	3.25	68.80	±	5.92	54.29	±	2.78	$p < 0.05$	A	A	B
6	66.18	±	3.46	81.96	±	5.20	53.95	±	2.99	$p < 0.05$	B	A	C
7	71.39	±	3.66	75.70	±	3.90	53.87	±	4.30	$p < 0.05$	A	A	B
8	73.19	±	4.72	71.37	±	3.10	53.40	±	2.88	$p < 0.05$	A	A	B
9	74.33	±	2.89	75.59	±	2.23	53.82	±	3.71	$p < 0.05$	A	A	B
10	74.46	±	3.86	81.19	±	9.26	57.37	±	3.16	$p < 0.05$	A	A	B
11	90.37	±	3.67	77.20	±	7.30	53.95	±	4.19	$p < 0.05$	A	A	B
12				85.58	±	3.56	57.93	±	11.28	NS			
13				87.45	±	3.28	60.65	±	5.42	$p < 0.05$		A	B
14							64.95	±	4.07				
