# Supplementary material for: Song Choice Is Modulated by Female Movement in Drosophila Males
Source: PLoS One. 2012 Sep 25;7(9):e46025. doi: 10.1371/journal.pone.0046025 (PMC3458092; doi:10.1371/journal.pone.0046025)
Supplement: Table S2 — Test results for total song production (Fig. S3). (PDF) [file pone.0046025.s006.pdf]

TableS2: ttest results for total song production (supplemental Fig3)

line crossing	ANOVA	WT	*Orco*$^{+}$	*Orco*^{2}
0	$p < 0.05$	B	A	AB
1	NS			
2	NS			
3	NS			
4	$p < 0.05$	B	B	A
5	$p < 0.05$	B	B	A
6	$p < 0.05$	B	B	A
7	$p < 0.05$	B	C	A
8	$p < 0.05$	B	C	A
9	$p < 0.05$	B	C	A
10	$p < 0.05$	B	B	A
11	$p < 0.05$	B	B	A
12	$p < 0.05$	B	B	A
13	$p < 0.05$	-	B	A
14	$p < 0.05$	-	B	A
